# Gene Silencing via PDA/ERK2‐siRNA‐Mediated Electrospun Fibers for Peritendinous Antiadhesion

**DOI:** 10.1002/advs.201801217

**Published:** 2018-11-20

**Authors:** Shen Liu, Fei Wu, Shanshan Gu, Tianyi Wu, Shun Chen, Shuai Chen, Chongyang Wang, Guanlan Huang, Tuo Jin, Wenguo Cui, Bruno Sarmento, Lianfu Deng, Cunyi Fan

**Affiliations:** ^1^ Department of Orthopaedics Shanghai Jiao Tong University Affiliated Sixth People's Hospital 600 Yishan Road Shanghai 200233 China; ^2^ Shanghai Key Laboratory for Prevention and Treatment of Bone and Joint Diseases Shanghai Institute of Traumatology and Orthopaedics Ruijin Hospital Shanghai Jiao Tong University School of Medicine 197 Ruijin 2nd Road Shanghai 200025 China; ^3^ School of Pharmacy Shanghai Jiao Tong University 800 Dongchuan Road Shanghai 200240 China; ^4^ Department of Pharmaceutical Sciences Laboratory Åbo Akademi University 20520 Turku Finland; ^5^ State Key Laboratory of Molecular Engineering of Polymers Fudan University No. 220 Handan Road Shanghai 200433 China; ^6^ I3S—Instituto de Investigação e Inovação em Saúde Universidade do Porto Rua Alfredo Allen, 208 Porto 4200‐135 Portugal; ^7^ INEB—Instituto de Engenharia Biomédica Universidade do Porto Rua Alfredo Allen, 208 Porto 4200‐135 Portugal; ^8^ CESPU—Instituto de Investigação e Formação Avançada em Ciências e Tecnologias da Saúde Rua Central de Gandra 1317 Gandra 4585‐116 Portugal

**Keywords:** adhesion prevention, adhesion tissue formation, electrospun fibers, gene delivery, siRNA

## Abstract

Sustained delivery of small interfering RNA (siRNA) is a challenge in gene silencing for managing gene‐related disorders. Although nanoparticle‐mediated electrospun fibers enable sustainable gene silencing, low efficiency, loss of biological activity, toxicity issues, and complex electrospinning techniques are all bottlenecks of these systems. Preventing peritendinous adhesion is crucial for their successful use, which involves blocking cellular signaling via physical barriers. Here, a multifunctional, yet structurally simple, cationic 2,6‐pyridinedicarboxaldehyde‐polyethylenimine (PDA)‐mediated extracellular signal‐regulated kinase (ERK)2‐siRNA polymeric delivery system is reported, in the form of peritendinous antiadhesion electrospun poly‐l‐lactic acid/hyaluronan membranes (P/H), with the ability to perform sustained release of bioactive siRNA for long‐term prevention of adhesions and ERK2 silencing. After 4 days of culture, the cell area and proliferation rate of chicken embryonic fibroblasts on siRNA+PDA+P/H membrane are significantly less than those on P/H and siRNA+P/H membranes. The in vivo results of average optical density of collagen type III (Col III) and gene expression of ERK2 and its downstream SMAD3 in the siRNA+PDA+P/H group are less than those of P/H and siRNA+P/H groups. Consequently, siRNA+PDA+P/H electrospun membrane can protect the bioactivity of ERK2‐siRNA and release it in a sustained manner. Moreover, adhesion formation is inhibited by reducing fibroblast proliferation and Col III deposition, and downregulating ERK2 and its downstream SMAD3.

## 1 Introduction

Gene silencing via small interfering RNA (siRNA) has revolutionized the downregulation of specific genes in recent years, with a potential value of billions of US dollars.^1^ Its rapid advancement offers new avenues, not only to investigate gene function and associated downstream signals, but also to block undruggable targets for the management of gene‐related disorders.^2^ Nevertheless, the therapeutic potential of siRNA can be impeded by premature serum degradation, renal clearance, and inefficient cell entry.^3^ As such, siRNA therapies have recently been designed using viral or nonviral vectors, mainly involving liposomes,^4^ cationic polymers,^5^ and peptide conjugation.^6^ Unfortunately, the transient silencing effects gradually became a main bottleneck in siRNA technology,^7^ so there is an urgent need for sustained release from carriers and a consequent increase of the bioavailability of siRNA.

Several studies have focused on nanofiber‐mediated delivery systems for the sustained availability of siRNA.^8^, ^9^ Such systems are constructed through surface immobilization or direct bulk incorporation of siRNA, with or without a transfection agent, in nanofibers.^10^, ^11^ Among these systems, nanoparticles, formed by the electrostatic self‐assembly of siRNA and loaded in electrospun fibers, are potentially promising for the provision of advanced sustainable gene silencing, but complex techniques of electrospinning are usually also required.^12^ Furthermore, when combined with electrospun membranes and a drug delivery system, siRNA may lose its functional activity through the interaction with heat, organic solvent, and the oil/water interface.^8^, ^13^ Moreover, certain types of nanoparticle, such as polyethylenimine (PEI), polyamidoamine dendrimers, polylysine, and chitosan, were shown to suffer from low efficiency and/or toxicity issues when incorporated into scaffolds.^14^ In this context, both the need to protect siRNA during electrospinning and the low transfection efficiency of nanoparticles may limit their physiological potential.

Peritendinous adhesion is one of the most common complications after tenolysis, resulting in severe limb disability.^15^ The pathology of adhesion involves the formation of peripheral adhesion tissues and subsequent invasion of adhesion tissues into the disrupted tendon.^16^ Recent efforts in this field have focused on antiadhesion tissue formation using electrospun fibrous membranes and membranes with drug agents to pharmacologically enhance antiadhesion ability.^17^ Owing to the high porosity and large aspect area of electrospun membranes, we previously incorporated ibuprofen and mitomycin‐C into electrospun fibrous membranes to prevent peritendinous adhesions.^18^, ^19^ However, as a challenge to overcome to achieve antiadhesion effects, adhesion tissue formation was not effectively blocked by these drug and fibrous combinations. It is now widely accepted that the successful inhibition of adhesion depends on blocking crucial cellular signaling and associated downstream signal pathways during adhesion formation.^20^ However, targeting the cellular signal that is crucial to the mechanism of adhesion is the key challenge here because this kind of targeting is usually referred to as blocking an undruggable gene without an inhibitor.

**Scheme 1:**
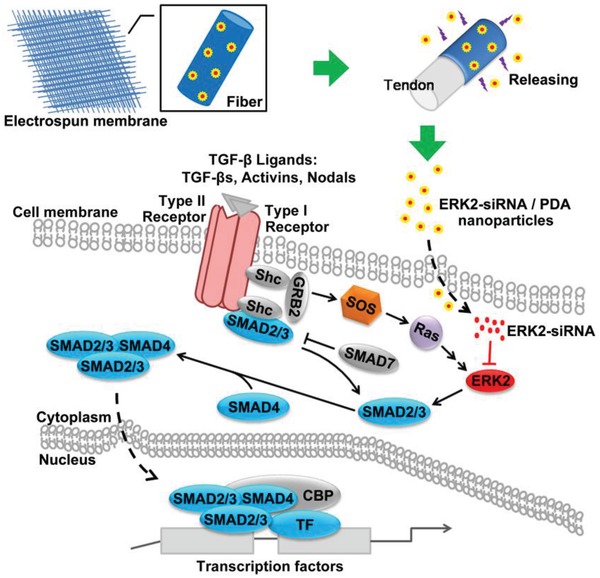
After incorporation of ERK2‐siRNA into PDA through an electrostatic self‐assembly process, an ERK2‐siRNA/PDA‐loaded PLLA electrospun fibrous membrane was fabricated by microsol electrospinning, producing a membrane that can sustainably release bioactive ERK2‐siRNA to block ERK2 and its downstream molecular signal.

We previously reported that silencing extracellular signal‐regulated kinase (ERK)2 can directly inhibit fibroblast proliferation and indirectly reduce collagen type III (Col III) deposition by the downregulation of SMAD2/3 in a model of rat knee capsular stiffness.^21^ Because both fibroblasts and Col III are the main components of peritendinous adhesion tissue, ERK2‐siRNA may block adhesion formation through a similar mechanism. In this study, we report on a multifunctional and structurally simple cationic polymer, 2,6‐pyridinedicarboxaldehyde‐polyethylenimine (PDA), and hypothesize that PDA‐mediated ERK2‐siRNA delivery systems in the form of peritendinous antiadhesion electrospun fibrous membranes can act as physical barriers preventing adhesions with the ability to perform the long‐lasting release of bioactive siRNA. It has also been postulated that PDA can protect bioactive ERK2‐siRNA by an electrostatic self‐assembly technique during microsol electrospinning for sustainably blocking ERK2 and its downstream SMAD3 signal to both achieve tissue separation and prevent adhesion formation (**Scheme** **1**). Here, the ERK2‐siRNA/PDA‐loaded poly‐l‐lactic acid (PLLA) membranes were characterized based on the antiproliferative effects in vitro, and the tissue‐separating and adhesion‐preventing effects in vivo.

## 2 Results

### 2.1 Characteristics of ERK2‐siRNA Polyplexes

**Figure 1:**
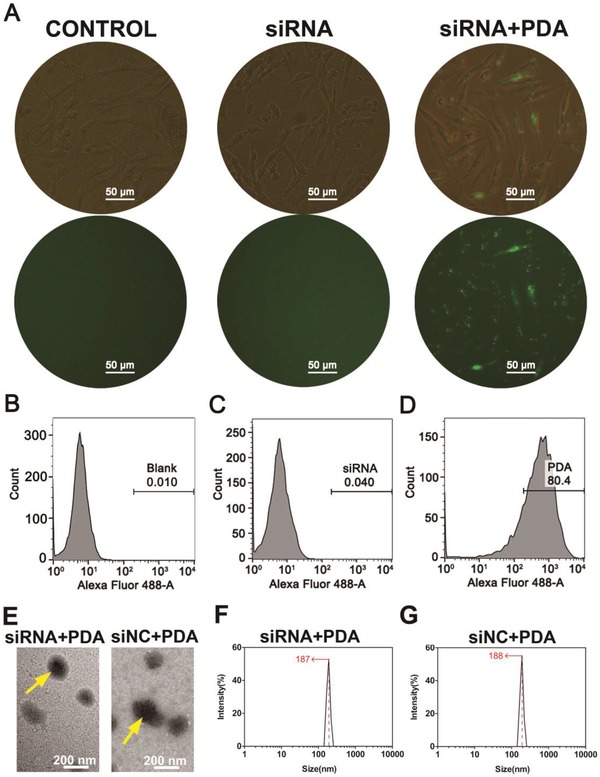
A) Fluorescent observation of transfected cells. B–D) Fluorescent observation of transfection efficiency as the percentage of 5′‐FAM‐positive cells determined by flow cytometry. E) TEM images of siNC/PDA and ERK2‐siRNA/PDA. F,G) Volume‐based distributions of polyplexes. CONTROL indicates blank control. * indicates *P* < 0.05.

The ERK2‐siRNA was effectively encapsulated into PDA polyplexes and harvested for in vitro transfection. The expression of 5′‐FAM reporter genes was detected in transfected chicken embryonic fibroblasts (UMNSAH/DF‐1) treated with ERK2‐siRNA/PDA polyplexes (**Figure** **1**A). By contrast, 5′‐FAM‐expressing cells could not be observed under a fluorescence microscope when treated simply with ERK2‐siRNA.

To quantify the transfection efficiency of ERK2‐siRNA/PDA polyplexes and ERK2‐siRNA, we determined the transfection efficiency for UMNSAH/DF‐1 cells using a flow cytometer. As shown in Figure 1B–D, the transfection efficiency of the ERK2‐siRNA/PDA group (w/w = 10) was nearly 80%, which is significantly higher than in the ERK2‐siRNA and blank control groups.

As shown in Figure 1E, the polyplexes were uniformly spherical with a smooth surface. The volume‐based distributions of the polyplexes were 187 ± 13 and 188 ± 15 nm for ERK2‐siRNA/PDA and small interfering negative control (siNC)/PDA polyplexes, respectively (Figure 1F,G).

### 2.2 Characterization of the Electrospun Fibrous Membranes

**Figure 2:**
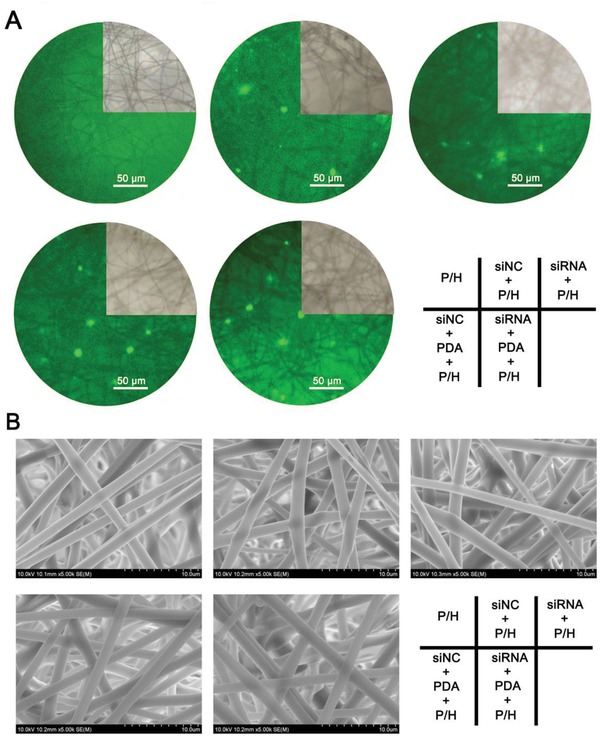
A) Fluorescent observation of P/H, siNC+P/H, siRNA+P/H, siNC+PDA+P/H, and siRNA+PDA+P/H membranes. B) The morphologies of P/H, siNC+P/H, siRNA+P/H, siNC+PDA+P/H, and siRNA+PDA+P/H membranes.

Fluorescent signals of siNC could be detected in the siNC‐loaded (siNC+P/H) and siNC/PDA‐loaded (siNC+PDA+P/H) PLLA/hyaluronan (HA) electrospun nanofibrous membranes, and for siRNA in the siRNA‐loaded (siRNA+P/H) and siRNA/PDA‐loaded (siRNA+PDA+P/H) PLLA/HA electrospun nanofibrous membranes, but not for P/H (**Figure** **2**A). Furthermore, green fluorescent particles were observed in the siNC+P/H, siRNA+P/H, siNC+PDA+P/H, and siRNA+PDA+P/H electrospun nanofibrous membranes, but not in the PLLA/HA electrospun nanofibrous membrane (P/H). Based on scanning electron microscopy (SEM) observation of their morphology, these electrospun fibrous membranes featured even fibers without beads (Figure 2B). The findings also revealed that these fibers were uniform in size, randomly interconnected in structure, and with a seemingly smooth surface.

**Figure 3:**
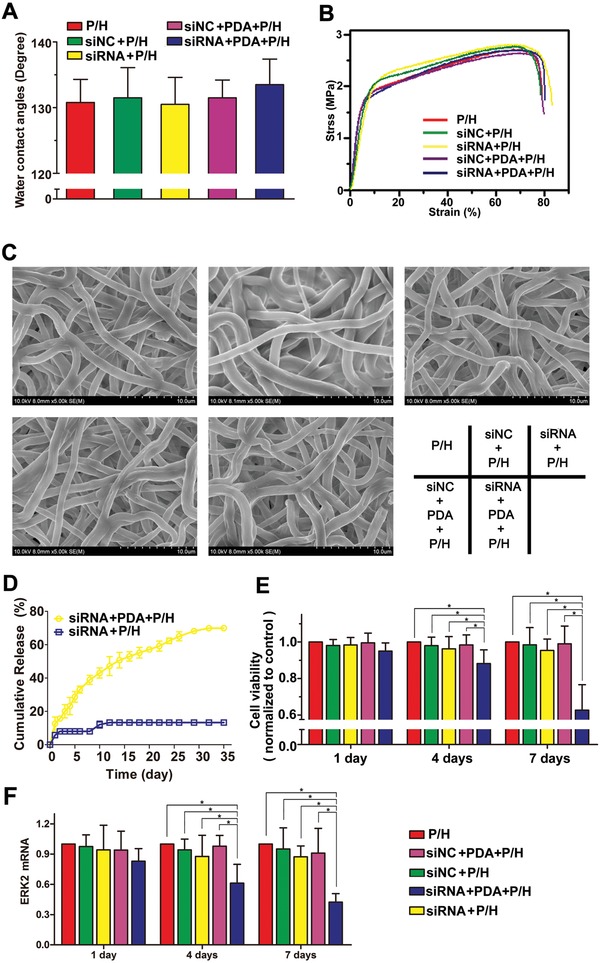
A) Water contact angles, B) stress/strain curves, C) degradation morphologies, D) in vitro ERK2‐siRNA cumulative release, E) proliferation analyses, and F) assessment of bioactivity of delivered gene for the P/H, siNC+P/H, siRNA+P/H, siNC+PDA+P/H, and siRNA+PDA+P/H membranes. * indicates *P* < 0.05.

The data on the characterization of the electrospun fibrous membranes are summarized in **Table** **1**. The diameters of the P/H, siNC+P/H, siRNA+P/H, siNC+PDA+P/H, and siRNA+PDA+P/H fibers were 1.62 ± 0.34, 1.97 ± 0.31, 1.92 ± 0.41, 2.02 ± 0.32, and 2.07 ± 0.33 µm, respectively. Furthermore, to clarify the surface properties of electrospun fibers, the water contact angles of P/H, siNC+P/H, siRNA+P/H, siNC+PDA+P/H, and siRNA+PDA+P/H membranes were measured as 131.6° ± 4.2°, 132.7° ± 5.5°, 130.1° ± 4.7°, 132.3° ± 3.7°, and 133.6° ± 4.1°, respectively, as shown in **Figure** **3**A.

**Table 1:** Characterization of the electrospun fibrous membranes

Name	Fiber diameter [µm]	Water contact angle [°]	Tensile strength [MPa]	Tensile moduli [MPa]
P/H	1.62 ± 0.34	131.6 ± 4.2	2.56 ± 0.25	24.25 ± 2.37
siNC+P/H	1.97 ± 0.31	132.7 ± 5.5	2.62 ± 0.29	23.54 ± 2.16
siRNA+P/H	1.92 ± 0.41	130.1 ± 4.7	2.68 ± 0.32	24.27 ± 1.82
siNC+PDA+P/H	2.02 ± 0.32	132.3 ± 3.7	2.52 ± 0.23	26.28 ± 2.24
siRNA+PDA+P/H	2.07 ± 0.33	133.6 ± 4.1	2.59 ± 0.24	27.17 ± 2.15

The mechanical properties of the different membranes were determined by creating a stress–strain curve, the results of which are shown in Figure 3B. The tensile strengths of P/H, siNC+P/H, siRNA+P/H, siNC+PDA+P/H, and siRNA+PDA+P/H membranes were 2.56 ± 0.25, 2.62 ± 0.29, 2.68 ± 0.32, 2.52 ± 0.23, and 2.59 ± 0.24 MPa, while their elastic moduli were 24.25 ± 2.37, 23.54 ± 2.16, 24.27 ± 1.82, 26.28 ± 2.24, and 27.17 ± 2.15 Mpa, respectively. These data show a trend of a decrease in the maximum elongation for the different electrospun fibrous membranes. However, in terms of the mechanical results, no significant differences were identified among the membranes.

### 2.3 In Vitro Release

The cumulative release curves of ERK2‐siRNA from siRNA+P/H and siRNA+PDA+P/H membranes are shown in Figure 3D. The cumulative release of ERK2‐siRNA from each membrane was calculated based on the daily release. The results showed that the siRNA+PDA+P/H membrane, in which ERK2‐siRNA was protected by PDA, exhibited no burst release of ERK2‐siRNA, followed by controlled release over 30 days, at the end of which cumulative release was more than 80%. However, siRNA+P/H exhibited rapid burst release of ERK2‐siRNA followed by a release kinetic less than 15 days, at the end of which the cumulative release of ERK2‐siRNA was less than 20%. The release of ERK2‐siRNA from ERK2‐siRNA/PDA‐loaded PLLA/HA membrane was relatively stable and lasted twice as long as that of the control group. The cumulative level of release of ERK2‐siRNA in the siRNA+PDA+P/H group was more than three times that in the siRNA+P/H group, indicating that the activity and integrity of ERK2‐siRNA were well protected in polyplexes by complexing with PDA.

After 20 days of incubation of the different membranes, SEM investigation revealed the integrity of their morphology and structure as physical barriers (Figure 3C). The level of degradation of the electrospun fibrous membranes was also determined by gravimetric analysis. The mass losses of P/H, siNC+P/H, siRNA+P/H, siNC+PDA+P/H, and siRNA+PDA+P/H membranes were 17.5 ± 3.8%, 16.8 ± 4.1%, 15.4 ± 3.4%, 16.1 ± 4.6%, and 18.1 ± 4.3% after incubation in phosphate‐buffered solution (PBS) for 20 days, respectively. There were no significant differences among these values.

### 2.4 In Vitro Cell Proliferation, Morphology, and Viability Analyses

The proliferation rate, intracellular uptake, and cell viability analyses were used to characterize the biological effects of siRNA released from membranes. The proliferation of UMNSAH/DF‐1 cells on the surfaces of P/H, siNC+P/H, siRNA+P/H, siNC+PDA+P/H, and siRNA+PDA+P/H membranes were compared at days 1, 4, and 7 (Figure 3E). The proliferation rate was statistically indistinguishable among these groups at day 1, but that of siRNA+PDA+P/H decreased significantly compared with those of the other four kinds of membranes at days 4 and 7. There was no statistically significant difference among the proliferation of P/H, siNC+P/H, siRNA+P/H, and siNC+PDA+P/H at the three time points.

The bioactivity of ERK2‐siRNA released from the membranes was determined using quantitative real‐time polymerase chain reaction (PCR), by showing knockdown of ERK2‐messenger RNA (mRNA) at days 1, 4, and 7 (Figure 3F). The expression levels of ERK2 mRNA showed no significant differences among these groups at day 1. Notably, the level of ERK2 in the siRNA+PDA+P/H group was significantly higher than those in the P/H, siNC+P/H, siRNA+P/H, and siNC+PDA+P/H groups at days 4 and 7. This indicates that the delivered ERK2‐siRNA was sufficiently bioactive to knock down the ERK2 expression over time.

**Figure 4:**
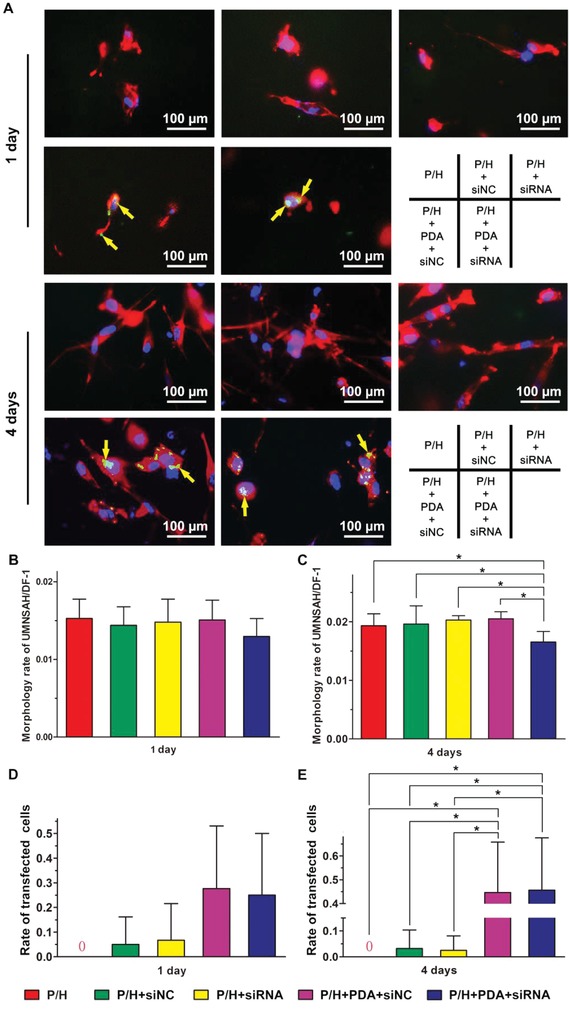
A) Intracellular uptake of drugs from the P/H, siNC+P/H, siRNA+P/H, siNC+PDA+P/H, and siRNA+PDA+P/H membranes. For each panel, nuclei were stained by 4′,6‐diamidino‐2‐phenylindole (DAPI) (blue); actin was stained by phalloidin (red); ERK2‐siRNA or siNC was labeled by 5′‐FAM (green). Antiadhesion ability of different membranes at B) 1 and C) 4 days after treatment was determined by CCK8 analyses. The proliferation rates of transfected cells at D) 1 and E) 4 days were determined by fluorescence microscopy. * indicates *P* < 0.05. Data are expressed as mean ± SD (each group, *n* = 3).

The adhesion of UMNSAH/DF‐1 cells could be detected on the surfaces of all of the different membranes (**Figure** **4**). Furthermore, the average cell area on each membrane was quantified using Photoshop 8.0 based on the scale bar in the associated images. The morphologies of UMNSAH/DF‐1 cells were better after 4 day culture than after 1 day culture. Although no significant difference was detected among the groups at day 1, the cells transfected with siRNA+PDA+P/H membrane showed a significantly lower average cell area at day 4. In particular, fluorophore 5′‐FAM‐labeled cells could be observed in the siNC+PDA+P/H and siRNA+PDA+P/H groups after 1 and 4 days of culture, although very little fluorescence was detected at day 1. The efficiency of siRNA transfection was calculated based on the fluorescence microscopy examination of 5′‐FAM‐labeled cells. At day 4, the transfection efficiencies of siNC+PDA+P/H and siRNA+PDA+P/H membranes were significantly higher than that of P/H, siNC+P/H, and siRNA+P/H membranes (Figure 4D,E).

**Figure 5:**
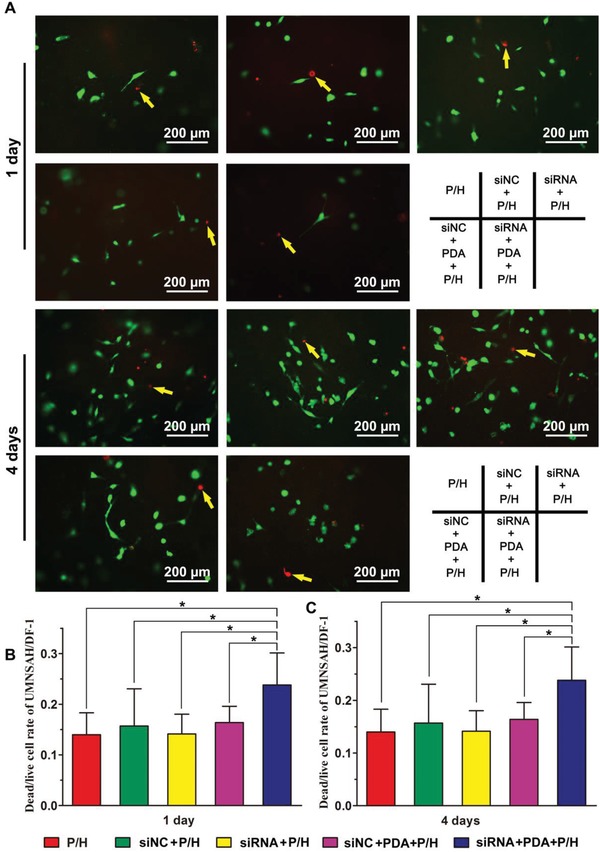
A) The results of a viability assay of cells grown on the surface of P/H, siNC+P/H, siRNA+P/H, siNC+PDA+P/H, and siRNA+PDA+P/H membranes. Dead/live rate of cells was determined at B) 1 and C) 4 days. * indicates *P* < 0.05. Data are expressed as mean ± SD (each group, *n* = 3).

The viability of UMNSAH/DF‐1 cells was investigated using their live/dead rate (**Figure** **5**). The numbers of both living and dead cells increased as time passed in each group, but the number of dead cells on the siRNA+PDA+P/H membrane increased markedly after 4 days of culture. Furthermore, regarding the viability results, the average dead/live rate of cells on the surface of siRNA+PDA+P/H at both time points was significantly higher than in the other four groups.

### 2.5 In Vivo Gross Observation

**Figure 6:**
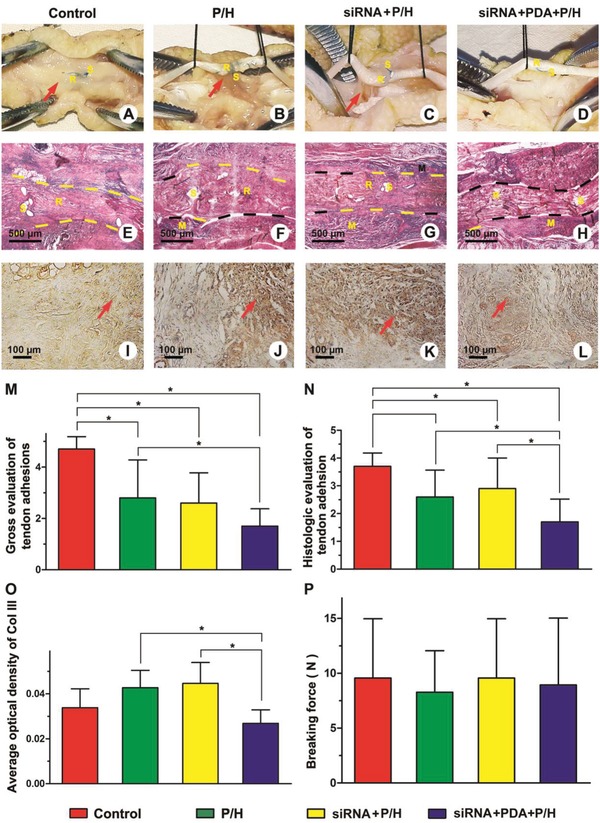
A–D) Gross evaluation of peritendinous adhesion of repaired sites after 21 days. E–H) Histological assessment of tendon adhesions. I–L) Immunohistochemical evaluation of Col III expression in peritendinous adhesion tissues. M) The gross scores and N) histological scores of peritendinous adhesion and O) average optical density of Col III in the peritendinous adhesion tissues. P) Tendon repair determined by the breaking force of the repaired tendons. R: repaired site; S: suture; M: residual materials. Black dotted lines indicate areas without adhesion; yellow dotted lines indicate adhesion areas. Red arrows indicate adhesion tissues in (A)–(C) and Col III‐positive tissues in (I)–(L). * indicates *P* < 0.05. Data are expressed as mean ± SD for 10 tendons per group.

After 21 days, no obvious ulcers or inflammatory exudates were detected on the toes. The repaired sites of the tendon were carefully examined to investigate the peritendinous adhesions directly (**Figure** **6**). In the control group without treatment, it was difficult to separate severe peritendinous adhesion tissues even by dissection using a scalpeat the repaired site (Figure 6A). In the groups receiving P/H and siRNA+P/H treatments (Figure 6B,C), the repaired sites were filled with dense bundles of adhesion tissues bridging between the repaired tendon and the peritendinous tissues, although parts of the adhesion areas could be released by separation with a blunt instrument. Almost no dense adhesion tissue formation was seen at the site of repaired tendon in the group receiving siRNA+PDA+P/H treatment. The parameters from the gross observation of adhesions are summarized in Figure 6N. The score of the siRNA+PDA+P/H group was the lowest among the four groups.

### 2.6 In Vivo Histological Assessments

The results of histological assessments of the peritendinous adhesions of repaired sites receiving different treatments are shown in Figure 6E–H. The peritendinous spaces of untreated tendons were filled with dense adhesion tissue. Meanwhile, adhesion tissue could be observed in repaired sites of the tendon with broken intratendinous collagen bundles (Figure 6E). Furthermore, some peritendinous adhesion tissues were detected on the epitenon surfaces of the repaired sites wrapped with P/H or siRNA+P/H membranes (Figure 6F,G). In the groups receiving siRNA+PDA+P/H treatment, clear obliteration of the peritendinous space was identified. Moreover, good continuity of the repaired site was identified, with a smooth epitenon surface. The peritendinous adhesions were significantly better in the siRNA+PDA+P/H group than in the other three groups (Figure 6O).

### 2.7 Analysis of Collagen III Deposition

Immunohistological staining was applied to analyze the location and density of Col III in the peritendinous adhesion tissues. Col III fibers stained brownish yellow were observed in all groups (Figure 6H–L). The Col III fibers in the peritendinous adhesion tissue of the siRNA+PDA+P/H group showed looser brownish yellow expression than those in the P/H group and the siRNA+P/H group. The Col III‐positive fibers showed no visible difference between the siRNA+P/H group and the P/H group. Considering the average optical density of Col III, the density of the siRNA+PDA+P/H group was significantly low among the four groups.

### 2.8 Biomechanical Analysis

The peak breaking forces, reflective of tendon healing, were recorded for mechanical analyses (Figure 6Q). The peak breaking force was found to be higher in the control group than in the other three groups treated with different membranes, although there were no significant differences among the four groups (Figure 6). These membranes were thus suggested to have no significant influence on tendon healing.

### 2.9 Protein Expression in Repaired Tendon

**Figure 7:**
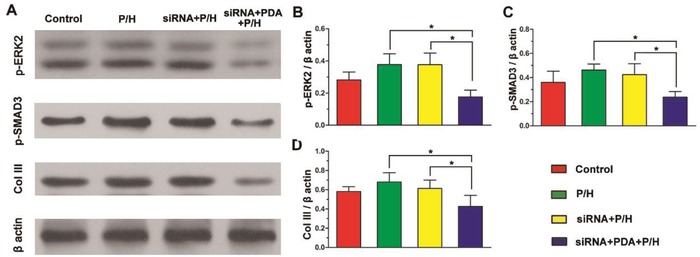
The expression of p‐ERK2, p‐SMAD3, and Col III in peritendinous adhesion tissues examined by western blotting, with A) β‐actin as a control. B) p‐ERK2, C) p‐SMAD3, and D) Col III expression normalized to β‐actin. * indicates *P* < 0.05.

To explore the cellular mechanisms behind the formation of adhesion tissue, the expressions of p‐ERK2, p‐SMAD3, and Col III in peritendinous adhesion tissues were examined by western blotting using β‐actin as a control (**Figure** **7**). The results showed that the expressions of p‐ERK2 and p‐SMAD3 were significantly lower in the group receiving siRNA+PDA+P/H treatment than in the other three groups (*P* < 0.05). Furthermore, the siRNA+PDA+P/H group showed the lowest Col III expression among the four groups, with a significant difference being identified (*P* < 0.05). The control group without implantation of membranes appeared to express less p‐ERK2, p‐SMAD3, and Col III than the P/H group and the siRNA+P/H group, but this did not reach significance.

## 3 Discussion

The formation of peritendinous adhesion tissue is a pathology related to fibroblast proliferation and Col III deposition. This study describes a PDA‐mediated ERK2‐siRNA delivery system in the form of electrospun membranes as a physical barrier for the long‐term prevention of adhesion formation. As shown by the obtained results, the controlled release of ERK2‐siRNA from the siRNA+PDA+P/H membrane was steady and lasted about twice as long as that of the siRNA+P/H membrane. Furthermore, as shown by our in vitro results, fewer UMNSAH/DF‐1 cells adhered to and proliferated on the siRNA+PDA+P/H membrane than on the P/H, siNC+P/H, siRNA+P/H, and siNC+PDA+P/H membranes due to the highly bioactive transfection of ERK2‐siRNA from the siRNA+PDA+P/H membrane. The in vivo study revealed that Col III deposition in the group receiving siRNA+PDA+P/H treatment decreased through downregulation of the expression of ERK2 and SMAD3 genes with the pronounced prevention of adhesion when compared with the other three groups.

Effective inhibition of the formation of peritendinous adhesions is related to the blocking of crucial cellular targets and associated downstream signals in the adhesion formation pathway.^22^ However, the mechanisms involved in peritendinous adhesion, especially crucial cellular targets, have not been clarified. Transforming growth factor (TGF)‐β1 plays critical roles in fibroblast proliferation and collagen expression.^23^ Some previous studies reported that TGF‐β can trigger the activity of SMAD and ERK, which are involved in fibrotic diseases, in certain cells such as human dermal fibroblasts, myofibroblasts, and cardiac fibroblasts.^24^ Furthermore, when ERK2 is downregulated, cellular proliferation is inhibited.^21^ In this study, the downregulation of ERK2 reduced the extent of peritendinous adhesion without significantly reducing tendon healing, as described in a previous study.^25^ This is probably because ERK2‐siRNA released from the siRNA+PDA+P/H membrane acted against the proliferation of fibroblasts, inhibited Col III deposition, and downregulated ERK2 expression and the level of its downstream factor SMAD3. These findings confirm that the targeting of ERK2 in downstream SMAD3 signaling plays a synergistic role in peritendinous adhesion formation.

Bioactive ERK2‐siRNA is critical for gene silencing. Commonly used nanoparticles such as PEI, polyamidoamine dendrimers, polylysine, and chitosan have been applied to protect nucleic acids, but they have not been widely used, possibly due to their low efficiency and toxicity issues.^26^ To overcome these issues, we previously established a type of PDA polyplex with outstanding gene transfection efficiency and negligible cytotoxicity when combined with pDNA, relative to PEI.^27^ However, the distinctive structural and chemical characteristics of DNA differ from those of RNA due to their respective complex formation properties.^28^ Against this background, the ability of PDA to facilitate siRNA delivery to silence a targeted gene should be investigated. Furthermore, the effect of PDA on maintaining the bioactivity of siRNA without denaturation is controversial during its encapsulation into physical barriers through the interaction with heat, organic solvent, and the oil/water interface. In this study, after PDA and siRNA were released from siRNA+PDA+P/H and PDA promoted the transfection of ERK2‐siRNA. However, ERK2‐siRNA from siRNA+P/H cannot pass the cellular membrane. Consequently, the antiproliferative efficiency of the siRNA+PDA+P/H membrane was significantly greater than that of the siRNA+P/H membrane. Therefore, PDA can not only promote targeted gene silencing, but also maintain siRNA bioactivity during the electrospinning process.

Recently, besides the development of novel carrier vectors, the main focus has been placed on modifying the currently available delivery systems.^8^, ^29^ Scaffold‐mediated RNA interference has been carried out through the surface immobilization or direct bulk encapsulation of siRNA in hydrogels,^30^ nanofibers,^11^ and sponge‐based constructs.^31^ For tissue separation, electrospun fibrous membranes allow the passage of nutrients from outside the tendon sheath to promote intrinsic healing based on their microporous structure.^19^, ^32^ Nevertheless, the development of electrospun barrier‐based siRNA therapeutics is still hampered by difficulties in achieving the sustained release of bioactive siRNA for long‐term applications. Recently, a few studies have used siRNA to increase the efficacy of adhesion barriers along with the prevention of adhesion formation. In this study, the efficiency of ERK2 release from the siRNA+PDA+P/H membrane reached 80%, which was completed within nearly 30 days, while that of siRNA+P/H membrane as a control was only 20%, which was completed within nearly 15 days. This may explain why the siRNA+PDA+P/H membrane was associated with significantly less formation of peritendinous adhesion tissue than the other three groups.

The present study has some limitations. Although the peak force of repaired tendon was investigated to reflect tendon healing, adhesion was not directly investigated mechanically. Further study should be performed to identify the difference in peritendinous adhesion determined mechanically compared with equivalent macroscopic and histological findings. Another limitation is based on the fact that, as revealed in our in vitro experiment, PDA cannot silence the ERK2 gene; only the siRNA of ERK2 itself can do this. However, no control group of siNC+PDA+P/H was included in the in vivo experiment. Without such a group, it cannot be definitively asserted that the results from the siRNA+PDA+P/H group are not due to the PDA. Thus, an siNC +PDA+P/H control group should be included in further in vivo studies. Moreover, future studies with features such as a longer experimental period and greater power for the statistical comparisons would be beneficial.

## 4 Conclusions

In this study, ERK2‐siRNA/PDA was fabricated and electrospun into PLLA/HA fibers. Using this delivery system, the cumulative release of ERK2‐siRNA from the siRNA+PDA+P/H membrane sustainably reached 80%, with a release time of nearly 30 days. The inhibition of cell proliferation and adhesion showed that the siRNA+PDA+P/H membrane can retain the biological activity of ERK2‐siRNA in a better suitable manner than the siRNA+P/H membrane. Moreover, the antiadhesion effect of the electrospun membranes as a barrier was determined as a clinical concern. Our results demonstrate that this siRNA+PDA+P/H membrane can protect the bioactivity of ERK2‐siRNA and is a promising platform to inhibit Col III deposition and downstream SMAD3 expression.

## 5 Experimental Section

*Materials*: PLLA (*M*_w_ = 50 kDa, *M*_w_/*M*_n_ = 1.61) was prepared by bulk ring‐opening polymerization of l‐lactide using stannous chloride as an initiator (Jinan Daigang Co., Jinan, China). Fermentation‐derived HA (sodium salt, *M*_w_ = 1.0 MDa), anhydrous ethylene dichloride (*M*_w_ = 1.8 kDa), and PEI (*M*_w_ = 25 kDa) were purchased from Sigma‐Aldrich Chemical Co. (St. Louis, MO) and used without further purification. Dichloromethane and trichloromethane were purchased from Chinese Medicine Group Chemical Reagent Corporation (AR). 2,6‐Pyridinedicarboxaldehyde (PD) was obtained from TCI Development Co., Ltd. (Shanghai, China). Three targeted sequences of ERK2‐siRNA (ERK2: 5′‐CAGUAGGCUGUAUUCUGGCAGAGAU‐3′) and small interfering negative control (siNC: 5′‐UUCUCCGAACGUGUCACGUTTACGUGACACGUUCGGAGAATT‐3′) were labeled with 5′‐FAM (GenePharma Co., Ltd., Shanghai, China). Cellulose membranes (*M*_w_ = 10 kDa) were purchased from Thermo Scientific. Poly(ethylene glycol) standard kit (*M*_w_ = 106 to 20 100 Da) was purchased from Polymer Standards Service GmbH. RNA assay kit was acquired from Invitrogen (Eugene, OR). Other chemicals and solvents were of reagent grade and purchased from Guoyao Reagents Company (Shanghai, China). All reagents were utilized without further purification.

*Preparation of ERK2‐siRNA‐Loaded PDA Polyplexes*: PDA was synthesized in accordance with a previously reported method.^27^ Briefly, 1 mmol PEI (*M*_w_ = 1.8 kDa) and 2 mmol PD were dissolved sequentially in a 20 mL solution of anhydrous ethylene dichloride. Then, PD solution was added dropwise into PEI solutions followed by stirring for 48 h at room temperature. After mixing, the solution was evaporated to retrieve the viscous residue. Subsequently, this residue was dissolved in deionized water and dialyzed using a cellulose membrane with a molecular weight cutoff of 10 000 Da for 24 h. A yellowish powder, representing PDA, was obtained after 2 days of lyophilization at −80 °C.

Next, ERK2‐siRNA/PDA polyplexes were obtained at a 1:10 weight/weight (w/w) ratio. Briefly, ERK2‐siRNA was dissolved in deionized water (20 ng µL^−1^), as was PDA (2 mg mL^−1^). Then, PDA stock solution was added to ERK2‐siRNA solution at the w/w ratio of 1:10 to induce the self‐assembly of polyplexes by pipetting. The samples were allowed to assemble at room temperature for 4 h. Similarly, siNC/PDA polyplexes (1:10, w/w) were prepared as a control.

*Characterization of ERK2‐siRNA/PDA Polyplexes*: The ERK2‐siRNA/PDA and siNC/PDA polyplexes were diluted in diethyl pyrocarbonate (DEPC) water and their particle size was measured using a Particle Size Analyzer (Brookhaven Instruments) at 25 °C. The morphology of ERK2‐siRNA/PDA and siNC/PDA polyplexes was observed by transmission electron microscopy (JEM 2010 system; JEOL).

*In Vitro Cell Transfection*: In vitro cell transfection of ERK2‐siRNA/PDA polyplexes in chicken embryonic fibroblasts (UMNSAH/DF‐1) was performed as described above. siRNA without PDA and normal culture medium were used as blank and negative controls.

The ERK2‐siRNA encoding 5′‐FAM was used to determine the transfection efficiency of ERK2‐siRNA/PDA or siNC/PDA polyplexes. The transfection of targeted ERK2‐siRNA/PDA polyplexes was observed under the fluorescence attachment of a Leica microscope (Leica Microsystems). A flow cytometer (BD FACSCalibur) was used to document the proportion of 5′‐FAM‐positive cells.

*In Vitro Gene Silencing*: UMNSAH/DF‐1 cells were purchased from the Cell Bank of the Chinese Academy of Sciences (Shanghai, China) and treated as a cell model for consistency with the in vivo study. These cells (5–10 × 10^4^ mL^−1^) were seeded in 24‐well plates and cultured in 1 mL of Dulbecco's modified Eagle medium (DMEM) containing 10% FBS and 1% antibiotics at 37 °C with a CO_2_ concentration of 5%. When the cells reached 80–90% confluence, the culture medium was changed to 200 µL of ERK2‐siRNA/PDA polyplex solution (w/w, 10:1) and 500 µL of DMEM medium for 4 h. Then, the culture medium was replaced with 1 mL of DMEM medium containing 10% FBS and 1% antibiotics for an additional 4 h. ERK2‐siNC/PDA polyplexes were treated as a negative control. Finally, they were observed under the fluorescence attachment of a Leica microscope (Leica Microsystems).

*Electrospinning of Fibrous Membranes*: Different electrospun fibrous membranes were fabricated using microsol electrospinning. Briefly, various solutions were first prepared. To prepare P/H, 1 g of PLLA was completely dissolved in a mixed solution of 4 g of dichloromethane, 2 g of *N*,*N*‐dimethylformamide, and 0.01 g of Span80; then, 200 mg of HA (1%, w/w) in H_2_O was added as stock solution with 10 min of stirring. To prepare siNC+P/H, 1 OD siNC was completely dissolved in 170 µL of DEPC solution and 0.8 mg of HA was subsequently added. To prepare siRNA+P/H, 150 µg of siRNA was completely dissolved in 170 µL of DEPC solution with 0.8 mg of HA subsequently added. To prepare siNC+PDA+P/H, 1 OD siNC was completely dispersed in 170 µL of DEPC solution and then 1.5 mg of PDA was added by stirring for 3 min. After sitting for 20 min at room temperature, 0.8 mg of HA was dissolved into the DEPC solution containing siRNA and PDA.

To prepare siRNA+PDA+P/H, 150 µg of siRNA was completely dissolved in 170 µL of DEPC solution with 1.5 mg of PDA subsequently added along with stirring for 3 min. After being allowed to stand for 20 min at room temperature, 0.8 mg of HA was added to the DEPC solution containing siNC and PDA. Then, the respective solutions were mixed with the stock solution by stirring for 10 min. Different solutions were drawn into glass syringes to prepare the fibers using a high‐voltage Statitron (Tianjing High Voltage Power Supply Co., Tianjin, China), whose maximal voltage was 30 kV.^33^ Subsequently, these solutions were used for electrospinning to fabricate core–shell fibers.

*Distribution of siRNA inside the Fibers*: The uniform distribution of siRNA inside the fibers was observed by monitoring the siRNA(encoding 5′‐FAM)‐loaded nanofibers under the fluorescence attachment of a Leica microscope (Leica Microsystems).

*Characterization of Morphology and Structure of the Electrospun Fibrous Membranes*: The morphologies of fibers were examined by field‐emission scanning electron microscopy (FESEM) using a Hitachi 4800 system with an acceleration voltage of 3.0 kV. Before SEM observation, the fibers were sputter‐coated with platinum. The static water contact angles of the microfibrous membranes were measured using a contact‐angle analyzer (DSA25S; Data Physics Corporation). The fibrous scaffolds were cut into strips (15.0 × 3.0 × 0.13 mm^3^) prior to mechanical tests using a mechanical testing machine (Shanghai Hengyi Precision Instruments Co., Ltd., Shanghai, China) at a speed of 0.5 mm s^−1^.

*In Vitro Degradation Study*: Different membranes were immersed in PBS to track their structural changes and mass loss due to degradation. After 20 days, the specimens were retrieved. The morphologies of the fibers after degradation were examined by FESEM as described above and the mass of the membranes was determined.

*In Vitro Release Study*: PBS (pH 7.4) was used for the in vitro release test. 5 mg of electrospun fibrous membrane was suspended in 1.0 mL of phosphate buffer (0.01 m, pH 7.4) in a 1.5 mL plastic vial (*n* = 3) to predict the release profiles under physiological conditions. The suspension was then incubated in an air rotator at 90 rpm and 37 °C for 35 days. At preset intervals, 0.2 mL of the supernatant was withdrawn and replaced by fresh buffer. The kinetics of release of ERK2‐siRNA in the electrospun fibrous membrane was measured using RiboGreen quantitative kit assay, following the manufacturer's protocol.

*In Vitro Cell Culture*: UMNSAH/DF‐1 cells were cultured as per previously described methods and further applied as a cell model for the evaluation of adhesion and proliferation. The cell density was 2 × 10^4^ cells cm^−2^ for the proliferation assay and 4 × 10^4^ cells cm^−2^ for the morphology and live/dead assays on different electrospun membrane surfaces at 37 °C with 5% CO_2_.^33^ Electrospun membrane discs with a diameter of 15 mm were sterilized with 75% ethanol in a 24‐well plate for 1 h and then washed twice with PBS for further studies.

*Cell Proliferation Assay*: The proliferation of UMNSAH/DF‐1 cells on the siNC+P/H, siRNA+P/H, siNC+PDA+P/H, and siRNA+PDA+P/H membranes was revealed by Cell Counting Kit‐8 (CCK8) analysis after 1, 4, and 7 days of cell culture, in accordance with the manufacturer's instructions. The P/H was used as a control. Briefly, 200 mL of CCK8 solution was added to each well of a 24‐well plate and incubated for 4 h. Subsequently, the absorbance of each specimen was documented at 490 nm using a spectrophotometer (Synergy 2; BioTek, Winooski, VT). The results are expressed using proliferation rate by normalizing the average absorbance value to that of cells on the P/H membrane at each respective time point.

*Assessment of Bioactivity of Delivered Gene*: The bioactivity of the delivered gene (ERK2‐siNC or ERK2‐siRNA) was assessed based on a quantitative real‐time polymerase chain reaction of ERK2. In brief, the cells were seeded on the siNC+P/H, siRNA+P/H, siNC+PDA+P/H, and siRNA+PDA+P/H membranes at a density of 4 × 10^4^ cells cm^−2^ at 37 °C with 5% CO_2_.^33^ At 1, 4, and 7 days after incubation, the quantitative real‐time PCR was performed using an Applied Biosystems ViiA 7 system (Applied Biosystems Inc., Foster City, CA, USA). The relative ERK2 mRNA levels in UMNSAH/DF‐1 cells on each membrane were evaluated, while glyceraldehyde‐3‐phosphate dehydrogenase (GAPDH) amplification was used as an internal control at the respective time points. RNA loading of each sample was normalized using GAPDH mRNA and subsequently used to analyze the relative expression levels of ERK2. The sequences of sense and antisense primers for related genes were as follows: ERK2: forward, 5′‐TGAAGACACAGCACCTCAGCAATG‐3′; reverse, 5′‐GGTGTTCAGCAGGAGGTTGGAAG‐3′; and GAPDH:forward, 5′‐TCCTGTGACTTCAATGGTGA‐3′; reverse, 5′‐CACAACACGGTTGCTGTATC‐3′. Relative quantification (*n* = 3) was applied using the comparative 2^−ΔΔCt^ method, as in a previous study.^34^

*Morphological Observation and Intracellular Uptake of siRNA*: The cytoskeleton and cell nucleus were observed using actin and DAPI staining, respectively, while the intracellular uptake of ERK2‐siRNA or siNC was determined using the 5′‐FAM fluorescence reporter gene after the incubation of UMNSAH/DF‐1 cells on different electrospun membranes for 1 and 4 days. Briefly, cells on the respective discs were first treated using 4% paraformaldehyde for 10 min. After washing twice with PBS, 0.1% Triton X‐100 (Sigma‐Aldrich) was then applied for 10 min. Subsequently, the cytoskeleton was stained using 20 µg mL^−1^ phalloidin (Sigma), in accordance with the manufacturer's instructions. Finally, the nucleus was stained with 1 µg mL^−1^ DAPI for 5 min and then observed under a confocal laser scanning microscope (Leica TCS SP2; Leica Microsystems, Heidelberg, Germany). Morphological observation and transfection efficiency are expressed using the average cell area and rate of fluorescent cells on the different membranes, respectively.

*Cell Viability Analyses*: Analyses of the viability of UMNSAH/DF‐1 cells on the different electrospun membranes were performed by dead/live staining using a Live/Dead stain kit (Invitrogen, Eugene, OR, USA) after 1 and 4 days of cell culture. Briefly, the cells on the different discs were stained with 2 × 10^−6^ m calcein AM and 10 × 10^−6^ m EthD‐1, and observed under a fluorescence microscope (DM 4000 B; Leica, Wetzlar, Germany) after 30 min of culture. The dead and living cells were stained red and bright green, respectively. The results are expressed using the dead/live cell rate.

*Animal Study*: All procedures of handling the experimental animals were performed in line with the policies of Shanghai Jiao Tong University School of Medicine after its permission. Leghorn chickens, weighing 1.5–2 kg, were used as an animal model of peritendinous adhesion. Briefly, after anesthetization (ketamine, 50 mg kg^−1^, intramuscular injection) and sterile skin preparation, a peritendinous adhesion model was established by repairing disrupted flexor digitorum profundus through a lateral skin incision on the proximal phalanx of the third toe using a modified version of Kessler tendon repair with a 6‐0 prolene suture (Ethicon Ltd., Edinburgh, UK).^33^ The animals were randomly assigned to four groups administered a 1 × 1 cm^2^ piece of: a) P/H, b) siRNA+P/H, c) siRNA+PDA+P/H, or d) no membrane. For the administration, the pieces were wrapped around the repaired site of the tendon and wound closure was performed with a 4‐0 silk suture (Ethicon Ltd., Edinburgh, UK).

*Macroscopic Evaluation*: At 3 weeks postoperatively, signs of inflammation or ulcers of each toe were evaluated. Then, the repaired sites were explored to categorize the severity of peritendinous adhesions using the adhesion scoring system, as described in our previous report.^35^ Grades 1–5 are quantified based on the surgical findings: grade 1, no adhesions on the tendon surface; grade 2, tiny adhesions can be easily separated by blunt dissection from the tendon surface; grade 3, less than or equal to 50% of the adhesion area is separable with difficulty using a blunt instrument but readily with a sharp one; grade 4, 51–97.5% of the adhesion area is only separable using a scalpel; and grade 5, more than 97.5% of the adhesion area is separable using a scalpel. The specimens were checked by two pathologists blinded to the experiments.

*Histological Evaluation*: At the determined time, the repaired sites of the tendon were retrieved and immersed in 4% paraformaldehyde for 1 day. After washing with PBS, specimens were completely decalcified in 10% ethylenediaminetetraacetic acid disodium salt and then embedded in paraffin through a graded series of ethanol. 4 µm sagittal sections were cut and stained with hematoxylin‐eosin. Associated histological scoring systems were used to examine adhesions and tendon healing.^36^ The histological scoring system of adhesions was used to grade the repaired sites into grades 1–4 as follows: grade 1, no adhesions on the repaired tendon; grade 2, adhesions constituting less than 33% of the area of the repaired site; grade 3, adhesions constituting 33–66% of the area of the repaired site; and grade 4, adhesions constituting more than 66% of the area of the repaired site.

*Immunohistochemical Evaluation*: Evaluation of Col III expression in peritendinous adhesion tissue was performed by immunohistological staining.^37^ Briefly, the prepared sections were dewaxed in xylene and subsequently hydrated in a graded series of ethanol. Then, they were heated in 10 × 10^−3^ m citrate buffer solution (pH 6.0) at 98 °C for 20 min for antigen recovery. Endogenous peroxidase activity was determined by incubation with 0.3% hydrogen peroxide while nonspecific sites were blocked using goat serum (1:100 dilution). Then, sections were treated with Col III antibodies (Abcam, Cambridge, MA, USA) overnight at 4 °C and subsequently with anti‐mouse rabbit antibodies for 1 h at 37 °C after repeated washes in PBS. Staining was developed using DAB solution (Dako, Hamburg, Germany), with counterstaining by hematoxylin.

*Biomechanical Evaluation*: To evaluate the tendon healing, the breaking strength of the repaired tendon was calculated using a rheometer (Instron 5548; Instron, Norwood, MA, USA).^19^ Briefly, the terminals of the repaired tendon were fixed to a force gauge and a custom‐made device with the proximal interdigital joint fixed by stainless‐steel rods. Then, the proximal ends were pulled at a rate of 20 mm min^−1^ until rupture to obtain a tension–elongation curve. Breaking force was recorded by a rheometer.

*Western Blot Analysis*: Western blotting was applied to investigate the phosphorylation of ERK2, SMAD3, Col III, and β‐actin.^37^ Briefly, peritendinous adhesion tissues were retrieved and homogenized in 100 µL of ice‐cold radio‐immunoprecipitation assay (Bio‐Rad, Hercules, CA, USA) supplemented with 1 µL of 100 × 10^−3^ m phenylmethanesulfonyl fluoride (Shen Neng Bo Cai Corp., Shanghai, China) for 30 min. Then, the protein concentrations of tissue lysates were quantified using bicinchoninic acid assay (Thermo, Rockford, IL, USA). The samples were separated by sodium dodecyl sulfate polyacrylamide gel electrophoresis, transferred onto polyvinylidene fluoride membranes (Millipore, Burlington, MA, USA), blocked in tris‐buffered saline and tween 20 containing 5% nonfat milk, and incubated with antibodies against p‐ERK2, p‐SMAD3, Col III, and β‐actin (diluted 1:1000; Abcam, Cambridge, MA, USA). Images were developed with an enhanced chemiluminescence reagent (Amersham Biosciences, Piscataway, NJ, USA) by the addition of the secondary antibodies (1:3000; Abcam, Cambridge, MA, USA). The results are expressed in terms of the p‐ERK2, p‐SMAD3, and Col III levels normalized to β‐actin bands by densitometry in Photoshop 8.0 (Adobe, San Jose, CA, USA).

*Statistical Analysis*: Data are expressed as mean ± standard deviation (SD). The statistical software SPSS 18.0 (Chicago, IL, USA) was used for one‐way analysis of variance followed by least‐significant difference post hoc tests. *p* < 0.05 was considered to represent a significant difference.

## Conflict of Interest

The authors declare no conflict of interest.

## Supporting information

Supplementary
