# Supplementary material for: Gene Silencing via PDA/ERK2‐siRNA‐Mediated Electrospun Fibers for Peritendinous Antiadhesion
Source: Adv Sci (Weinh). 2018 Nov 20;6(2):1801217. doi: 10.1002/advs.201801217 (PMC6343062; doi:10.1002/advs.201801217)
Supplement: Supplementary file 1 — Supplementary [file ADVS-6-1801217-s001.pdf]

Copyright WILEY-VCH Verlag GmbH & Co. KGaA, 69469 Weinheim, Germany, 2018.

ADVANCED SCIENCE
Open Access

Supporting Information

for *Adv. Sci.,* DOI: 10.1002/advs.201801217

Gene Silencing via PDA/ERK2-siRNA-Mediated Electrospun Fibers for Peritendinous Antiadhesion

Shen Liu, Fei Wu, Shanshan Gu, Tianyi Wu, Shun Chen, Shuai Chen, Chongyang Wang, Guanlan Huang, Tuo Jin, Wenguo Cui, Bruno Sarmento,* Lianfu Deng,* and Cunyi Fan**

DOI: 10.1002/ ((please add manuscript number))

Gene Silencing via PDA/ERK2-siRNA Mediated Electrospun Fibers for Peritendinous Anti-adhesion

Shen Liu, Fei Wu, Shanshan Gu, Tianyi Wu, Shun Chen, Shuai Chen, Chongyang Wang, Guanlan Huang, Tuo Jin, Wenguo Cui*, Bruno Sarmento*, Lianfu Deng*, and Cunyi Fan*

Dr. S. Liu, T Wu, S Chen, C Wang, Prof. C. Fan,
Department of Orthopaedics, Shanghai Sixth People’s Hospital, Shanghai Jiao Tong University School of Medicine, 600 Yishan Road, Shanghai 200233, China
E-mail: cyfan@sjtu.edu.cn
Prof. L Deng, Prof. W Cui
Shanghai Institute of Traumatology and Orthopaedics, Shanghai Key Laboratory for Prevention and Treatment of Bone and Joint Diseases, Ruijin Hospital, Shanghai Jiao Tong University School of Medicine, 197 Ruijin 2nd Road, Shanghai 200025, China
E-mail: lf_deng@126.com; wgcui80@hotmail.com
Dr. F Wu, S Gu, S Chen, T Jin
School of Pharmacy, Shanghai Jiao Tong University 800 Dongchuan Road, Shanghai 200240, China
Prof. B Sarmento
I3S - Instituto de Investigação e Inovação em Saúde, Universidade do Porto, Rua Alfredo Allen, 208, Porto 4200-135, Portugal
INEB - Instituto de Engenharia Biomédica, Universidade do Porto, Rua Alfredo Allen, 208, 4200-135, Portugal
CESPU - Instituto de Investigação e Formação Avançada em Ciências e Tecnologias da Saúde, Rua Central de Gandra 1317, Gandra 4585-116, Portugal
E-mail: bruno.sarmento@ineb.up.pt
Guanlan Huang, M.D.
Shanghai Jiao Tong University School of Medicine, 227 South Chongqing Road, Shanghai 200025, China

Table S1. Characterization of the electrospun fibrous membranes.

**Name**	**Fiber diameter (μm)**	**Water contact angle (°)**	**Tensile strength (MPa)**	**Tensile moduli (MPa)**
P/H	1.62 ± 0.34	131.6 ± 4.2	2.56 ± 0.25	24.25± 2.37
siNC+P/H	1.97 ± 0.31	132.7 ± 5.5	2.62 ± 0.29	23.54± 2.16
siRNA+P/H	1.92 ± 0.41	130.1 ± 4.7	2.68 ± 0.32	24.27± 1.82
siNC+PDA+P/H	2.02± 0.32	132.3 ± 3.7	2.52 ± 0.23	26.28± 2.24
siRNA+PDA+P/H	2.07 ± 0.33	133.6 ± 4.1	2.59 ± 0.24	27.17± 2.15
